# The impact of communication education provided with creative drama method on midwifery undergraduates

**DOI:** 10.18332/ejm/138592

**Published:** 2021-09-24

**Authors:** Arzu Kul Uçtu, Nazan Karahan

**Affiliations:** 1 Midwifery Department, Gulhane Faculty of Health Sciences, University of Health Sciences, Ankara, Turkey

**Keywords:** creative drama, midwifery, midwifery education, communication, self-esteem

## Abstract

**INTRODUCTION:**

This study was carried out to evaluate the impact of communication education provided by using creative drama method on the communication skills, selfesteem, and organizational conflict resolution skills of midwifery undergraduates.

**METHODS:**

The research was conducted as a semi-experimental ‘controlled pretest–posttest’ method. The study was carried out with freshmen studying midwifery at a public university in Western Turkey (n=52) between 30 September and 30 December 2017. Data were collected by means of the Communication Skills Inventory, Rosenberg Self-Esteem Scale, and Rahim Organizational Conflict Inventory-II. Throughout the research process, a 12-week lesson plan covering the stages of the creative drama method was applied. During the collection of the data, the scales were applied to the group as pretest, posttest while dependent t-test was applied for analyzing purposes.

**RESULTS:**

Communication Skills Inventory behavioral communication skills created a significant difference between Rosenberg Self-Esteem Scale self-esteem subscale scores (p>0.05). No significant difference was detected among the Rahim Organizational Conflict Inventory sub-dimension mean scores (p>0.05).

**CONCLUSIONS:**

Findings obtained from the research reveal that the creative drama method effectively develops communication skills, self-esteem, and conflict resolution skills for midwifery undergraduates.

## INTRODUCTION

The primary condition for providing high-quality midwifery care is to have well-trained midwives. The report entitled ‘Midwifery 2020’ highlights that the vision of midwifery education is to ‘turn midwives into reliable and autonomous practitioners’. To put this vision into practice, midwifery education needs to be run competence-based rather than as an academic degree, in line with the recommendations of the International Confederation of Midwives (ICM). ICM defines this competence as ‘putting the knowledge, skills and conducts securely and effectively into action without the need for monitoring or supervision’ and to ensure the competence of a practice, it underlines that critical-thinking, clinical decision-making, problem-solving and interpersonal communication skills be improved in addition to knowledge and conducts^1^.

The cognitive skills of individuals are closely related to their self-esteem. Self-esteem is connected to one’s inner valence and a concept affecting the whole life^2,3^. Self-esteem is necessary to establish and maintain social and emotional well-being. It is reported that those with high self-esteem are making their way better at demonstrating their potential, taking charge in important positions and maintaining interpersonal communication skills^4,5^. On the other hand, those with low self-esteem have low decision-making skills, and they are often in need of others’ impulsions, lack rationality, and experience negative emotions such as hostility and fear^6,7^. People with low self-esteem tend to go through psychological problems and self-blame for mistakes in addition to being in need of others’ approvals^6,8,9.^ Self-esteem has an impact on one’s intrafamilial relations, educational background, choice of profession and success in the practice of that profession, in short, their entire life^2^.

With all these taken into account, midwives need to have high self-esteem. All efforts to increase the capacity of midwives’ self-esteem could contribute to the development of cognitive skills of midwifery undergraduates. Considering that upskilling critical thinking, problem-solving and clinical decision-making are rather difficult at midwifery education^10^, it gains prominence to benefit from up-to-date educational methods before graduation through demonstrations and simulations by means of case studies^11^.

One of the current methods to equip the students with cognitive skills is creative drama. It is defined by San^12^ as ‘the individuals interpreting and animating an experience, an incident, an idea or sometimes an abstract concept by means of game-like processes in group work and by drawing on such theatrical techniques as improvisation and role-playing’. Any subject of a class is possible to be discussed using the creative drama method. Despite the fact that creative drama experiences are dependent on the study of the leader, it is generally made up of preparatory-warmup, interpretation, and evaluation phases^13^. Drama activity offers the utmost benefit in learning activities as it engages logically creative activities^12,14^.

Creative drama activities provide the participants with the opportunities to introduce themselves, to be able to put themselves in others’ position and with the facility to discover everyday life incidents during creative processes^13^. The studies demonstrate that students improve their self-esteem, increase their awareness about being a group member, and develop their communication and problem-solving skills^14,15^.

During the literature review, no information was found concerning the previous experiences of midwifery undergraduates on learning with creative drama techniques, nor was a previous study identified about analyzing the efficiency of creative drama technique in midwifery undergraduates acquiring cognitive skills. Therefore, our study is rather unprecedented. The purpose of this research is to assess the impact of applying creative drama as the method of communication education for midwives on self-esteem and organizational conflict solving skills.

### Hypotheses

1. There is a statistically significant difference between mean scores of subscales and overall mean scores of the Communication Skills Inventory of pre and post creative drama course of midwifery undergraduates.
2. There is a statistically significant difference between mean scores of subscales and overall mean scores of the Rosenberg Self-Esteem Inventory of pre and post creative drama course of midwifery undergraduates.
3. There is a statistically significant difference between mean scores of subscales and overall mean scores of the Rahim Organizational Conflict Scale of pre and post creative drama course of midwifery undergraduates.

## METHODS

### Study design

Our study design is an experimental study based on the ‘single group pretest-posttest’ design model. In accordance with this research model, the students were assessed one time before applying the creative drama activities and another time following the completion of education modules prepared via creative drama techniques. The study was carried out at a Midwifery Department of Health Sciences Faculty of a university which is located in western Turkey between 30 September and 30 December 2017.

### Population and sampling

The study population is composed of freshmen following undergraduate studies at the Midwifery Department of Health Sciences Faculty of The University during 2017–2018 academic year (n=76). Sampling was not realized for this research, and convenience sampling was applied. The study group of research comprised 52 volunteering students who were actively enrolled in undergraduate studies. Those students who declined to participate in the research and those who did not follow at least two classes were excluded. Additionally, 8 students who initially agreed to take part in the study, did not regularly follow the sessions and were excluded from the analysis.

### Data collection tools

Data of the research were collected by using Communication Skills Inventory, Rosenberg Self-Esteem Scale and Rahim Organizational Conflict Inventory-II.

*Communication Skills Inventory*

Developed by Balcı (1996) and reviewed by Ersanlı and Balcı (1998), it comprises 45 questions with a Likert-type scale. Items were answered as: ‘always’, ‘generally’, ‘sometimes’, ‘seldom’, and ‘never’. Scale is constituted by 3 sub-dimensions: mental, emotional, and behavioral. Each sub-dimension consists of 15 items. Those items belonging to the mental dimension are the questions numbered: 1, 3, 6, 12, 15, 17, 18, 20, 24, 28, 30, 33, 37, 43 and 45. Those belonging to the emotional dimension are the questions numbered: 5, 9, 11, 26, 27, 29, 31, 34, 35, 36, 38, 39, 40, 42 and 44. The questions measuring the behavioral dimension were: 2, 4, 7, 8, 10, 13, 14, 16, 19, 21, 22, 23, 25, 32 and 41. The highest score that can be obtained from the overall scale is 225, whereas the lowest score is 45. As per the scores obtained from the scale, the higher the scores obtained from the overall scale or its sub-dimensions, the better the communication skills.

*Rosenberg Self-Esteem Scale*

Developed by Morris Rosenberg (1963), it has been validated and authenticated in Turkish by Çuhadaroğlu (1985). Commonly used in studies, the scale consists of 12 sub-dimensions: covering self-esteem, continuity of the concept of self, trusting people, sensitivity to criticism, depressive affect, fancifulness, feeling threatened in interpersonal relations, participation in discussions, psychic isolation, psychosomatic symptoms, and parental interest. Only the self-esteem sub-dimension of the scale was applied in the present study. The self-esteem sub-dimension of the scale comprises a total of 10 items, including positive-negative statements. The scores obtained from the scale are interpreted as follows: 0–1 as high, 2–4 as medium, and ≥5 as low self-esteem. Hence, the higher the numbers scored by individuals the lower their self-esteem.

*Rahim Organizational Conflict Inventory-II*

This inventory was developed by Rahim in order to measure five independent styles of managing interpersonal conflicts^16^. Translated into Turkish by Kozan and Ilter (1994), the acceptable credibility of the inventory was also established. The scale is made up of 28 items and has a five-point Likert-type scale and scored as: do not agree (1 point) and totally agree (5 points). The scale consists of 5 sub-dimensions covering integration, domination, avoidance, compliance, and compromise. The dimension scoring the highest points among those making up the scale indicates the strategy used in the conflict by the relevant party.

### Application of the research

Between the years 2010–2020 Republic of Turkey Ministry of Education Special Doğaç Drama six stages of training of creative drama leadership was completed by one of the authors. The study was carried out by researchers with creative drama application certification. The research was performed in three stages. At the first stage, the students were informed about the importance and objective of the research and provided consent to participate in the study. Afterwards, all students were provided with data collection forms. The research was conducted with the approval of the Non-Interventional Ethics Committee of The University (Date of decision: 26/04/2017 Decision No: 4/42).

At the second stage of the research a communication class was carried out with the creative drama technique. Students volunteering for the research (n=52) were divided into 3 groups composed of 17–18 people chosen randomly. Each group received a 12-week communication skills educational program with a weekly duration of 90 minutes. Such techniques as role-playing, writing in the role, role card, frozen image, split screen, reversal, improvisation, meeting technique, and forum theater, were implemented during the program. For each session warm-up games related to communication, trust or conflict and problem solving were played in line with the pre-determined gains. Session assessments were conducted with activities increasing thinking such as group discussions, questions and answers, and brain storming. Weekly creative drama education sessions were structured in line with the literature, as given below. All sessions were moderated by a lecturer holding a creative drama leadership certificate. Contents were enriched with examples specific to the midwifery profession (Table 1). At the last stage of research, one week after the completion of the 12-week creative drama communication education, the scales were applied once more.

**Table 1:** Sessions of the educational program

*Session*	*Subject*
1	Introduction and communication
2	Trust and compliance activities
3	Perception and emotion activities
4	Self-acquaintance
5	Recognizing emotions
6	Effective communication
7	Obstacles to communication
8	Self-conception
9	Self-esteem
10	Conflict solving skills
11	Teamwork and communication
12	Teamwork and problem solving

### Evaluation of data

The normal distribution of the data was analyzed with a Kolmogorov-Smirnov test. Accordingly, the significance value was greater than 0.05 in the data obtained in the study, the value of steepness and skewness was between -1 and +1, and the data were normally distributed. Therefore, parametric tests were used in the analysis. The difference between the pretest–posttest scale mean scores was evaluated by applying a t-test on the dependent groups.

## RESULTS

The mean age of the students was 18.8±4.8 years, the marital status of all was single, 82.7% graduated from high schools outside vocational high schools and 48.1% spent most of their life in metropolitan areas. Comparison of the communication skills inventory scores and sub-dimension mean scores applied pre and post creative drama education of the undergraduates is given in Table 2.

**Table 2:** Results relating to pretest–posttest mean scores of communication skills inventory

*Communication skills inventory*		*Mean score*	*SS*	*t*	*p*
**Mental communication skills**	Pre	30.48	6.68±0.92	0.579	0.565
	Post	31.21	5.22±0.725		
**Emotional communication skills**	Pre	37.78	5.75±0.79	1.22	0.225
	Post	39.23	7.09±0.98		
**Behavioral communication skills**	Pre	32.36	4.83±0.67	3.22	**0.02**
	Post	36.21	7.04±0.97		
**Overall**	Pre	100.63	13.12±1.82	2.86	0.06
	Post	109.57	18.36±2.5		

The mean score of the students’ (n=52) pre-education Communication Skills Inventory was 100.63±13.12, and the mean for post-education was 109.57±18.39. There was a significant difference between the behavioral communication skills sub-dimension scores of the students Communication Skills Inventory pre and post creative drama course (p=0.02). Although the Communication Skills Inventory increased pre and post education, emotional communication skills, behavioral communication skills and scale mean scores, the difference was not significant (p>0.05) (Table 2).

A significant difference was identified between self-esteem subscale scores of the Rosenberg Self-Esteem Scale pre and post students’ creative drama education (p=0.02) (Table 3).

**Table 3:** Comparison of Rosenberg Self-Esteem Scale pretest-posttest mean scores

*Rosenberg Self-Esteem Scale*		*Mean score*	*SS*	*t*	*p*
**Self-esteem**	Pre	5.45	5.01±0.69	3.35	**0.02**
	Post	3.07	0.78±0.10		

**Figure 1:**
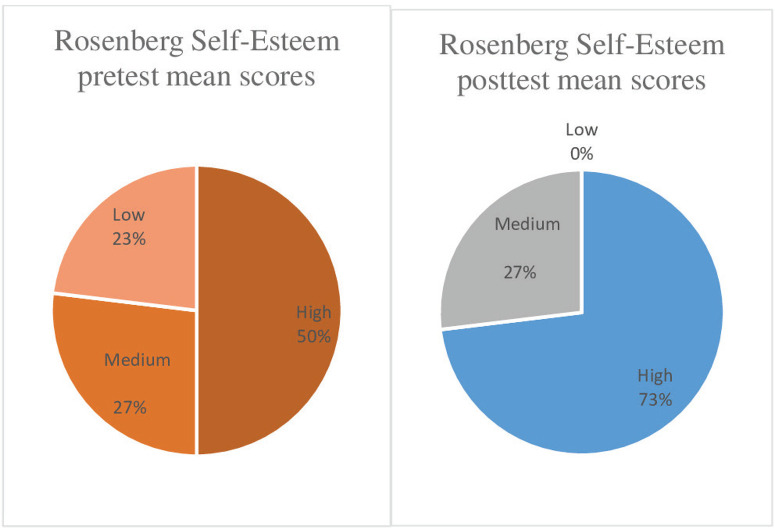
Self-esteem levels of students as per the Rosenberg Self-Esteem Scale, pre and post creative drama education

In accordance with Rosenberg Self-Esteem Scale, the self-esteem sub-dimension of the students before the creative drama education, the self-esteem levels were 50% high, 27% medium, and 23% low. Following the creative drama education, according to the Rosenberg Self-Esteem Scale self-esteem sub-dimension, 73% had high and 27% had medium self-esteem (Figure 1).

The students who participated in the study used the most avoidance (17.17±2.04) management style and applied the least compromise (6.82±4.56) management style in managing their conflicts with their peers before education. Post-education avoidance management style (16.26±2.17) decreased in the mean score, and no significant difference was found between the mean scores (p=0.745) (Table 4).

**Table 4:** Comparison of Rahim Organizational Conflict Inventory-II pretest and posttest mean scores

*Communication skills inventory*		*Mean score*	*SS*	*t*	*p*
**Integration**	Pre	11.50	2.42±0.33	0.612	0.543
	Post	11.82	2.73±0.37		
**Compliance**	Pre	14.05	3.02±0.41	1.139	0.260
	Post	14.80	3.39±0.47		
**Domination**	Pre	13.05	3.58±0.53	0.641	0.524
	Post	12.57	3.40±0.47		
**Avoidance**	Pre	17.17	2.04±0.28	0.328	0.745
	Post	16.26	2.17±0.30		
**Compromise**	Pre	6.82	4.56±0.63	0.993	0.326
	Post	6.96	3.91±0.54		

## DISCUSSION

Midwifery is a social profession intermingled with individual, family and society. For this reason, midwives actively make use of their communication and problem-solving skills while performing their profession^17,18^. Communication skills, self-esteem and techniques used in conflict resolution practiced in midwifery are of significance with regard to the service quality^19,20^. To improve the caring quality, it is essential that the communication problems healthcare workers experience with people they care be eliminated as one of the principal secure and low-cost methods^21^.

Midwifery undergraduates’ acquisition of effective communication skills is necessary for them to follow a successful educational life and become professional midwives. However, the classical approach used in formal education offers students a passive position and influences their communication skills acquisition process negatively. Moreover, it is common knowledge that equipping midwives with cognitive skills is rather difficult and time-consuming^10^. Thus, it will be advantageous to utilize up-to-date educational methods, such as creative drama, with the aim of investing in cognitive skills. This study was carried out with 52 midwifery freshmen to evaluate the effect of using creative drama as a method in education in midwifery communication skills, self-esteem and organizational conflict resolution skills.

In our study, there was an increase in the Communication Skills Inventory total scores of the midwifery undergraduates (n=52) pre and post creative drama course and a statistically significant increase in the behavioral communication skills sub-dimension.

Creative drama is an interdisciplinary teaching method based on the learning by living, using the body and all sensory organs. Creative drama affects cognitive, sensory and psychomotor areas and augments life-long learning^13,22^. It was demonstrated through various studies that the creative drama method, which can be applied for all ages, starting from pre-school period, contributes to the acquisition of communication skills. A study conducted with students of Turkish teaching department, determined that the students who received communication education by means of creative drama improved their skills of initiating communication and using effective communication methods^23^. In a semi-empirical study conducted to examine the effect of providing creative drama-based interpersonal relations education to university students on communication and social problem-solving skills, students were classified into: those who received drama-based communication education, those who did not receive it; and those who received education on the contents except for communication courses via creative drama methods and those who did not receive it. The results of this study proved that communication skills and social problem solving skills of the students participating in the Creative Drama-Based Interpersonal Relations education program are statistically significantly higher than those who did not take creative drama education and those students who took education on the contents except for communication courses via creative drama methods^24^. Another study conducted with the students of a science education department indicated that creative drama education is an effective method for the solution of communication problems, and the change that teacher candidates experience during their education will guide them in terms of using the method in their professional lives^25^. According to a study examining the use of creative drama method in the development of critical thinking skills of nursing students it was reported that creative drama is effective and should be included in the curriculum in strengthening critical thinking skills of healthcare professionals^26^. As a result of a study evaluating the impact of creative drama method on the adoption of professional values for midwifery students, it was found that creative drama facilitates students to learn professional values. Furthermore, in addition to the development of professional values, the study set out that the knowledge experienced by living affects positively the communication with pregnant women during the practices^27^. There are various practice examples in the literature that can improve empathic skills for midwives^28^ and nurse students^29^. However, no examples of studies that develop creative drama-based communication skills have been encountered. Our study finding suggests that creative drama empowers the communication skills of midwifery undergraduates, which is in accordance with the literature.

High level of self-esteem is important for midwives to fulfill their professional roles. In our study, there was a statistically significant increase in the self-esteem subscale scores of the Rosenberg Self-Esteem Scale pre and post creative drama course.

Creative drama is a method that contributes significantly to the evolvement of one’s self-esteem. Creative drama helps individuals increase their self-esteem by knowing themselves in their professional and social lives, recognizing their competencies, developing a sense of appreciation or approval^27^. A study conducted to evaluate the effects of creative drama course on child development department students’ (n=87) self-esteem established that the scores of self-perception showed a statistically significant difference and that creative drama education enabled the increase of self-perception^22^. During a systematic review examining the use of creative drama in nursing education, it was reported that creative drama helps students express themselves comfortably, contributes to the development of self-esteem and problem solving skills^30^. No studies investigating the effect of creative drama on students’ self-esteem scores were available. Our study finding shows that, in accordance with the literature, the use of creative drama method within the communication course enhances the self-esteem of midwifery undergraduates and suggests that the creative drama method could be a good tool for them to gain cognitive skills.

Improving conflict resolution skills of midwifery undergraduates contributes in a positive way to maintaining interpersonal relationships effectively and to the solution of professional problems they may encounter^31^. Increasing the self-esteem of midwifery undergraduates who are to work in institutional areas such as hospitals and team work with other healthcare workers will have an impact on the way they find a solution to problems and conflicts^6^. Studies point out that individuals with low self-perception use avoidance and compliance style as conflict resolution styles^31,32^ while those with high levels of self-perception tend to utilize integration style^33^. In our study conducted for conflict management purposes, it was designated that students used avoidance form the most and compromise form the least before taking creative drama education, and following the creative drama education, the avoidance of conflict had a decrease, yet no significant difference was observed between the sub-dimensions of the scale pre and post education.

Even though in the literature there are a many studies indicating that the creative drama technique has a positive effect on conflict resolution skills in children and adolescents^30^, no studies were encountered investigating the effect of creative drama in developing conflict resolution behavior in adults. Creative drama is a method that offers individuals the feeling of belonging to a group and enables effective communication techniques to be engaged in solving problems and conflicts. Nevertheless, it is a time-consuming process for education to result in behavioral change for adults^34^.

The program, which was conducted for 12 weeks throughout our research, triggered a change in the organizational conflict inventory avoidance and reconciliation sub-dimension mean scores. However, this change did not indicate a statistically significant difference. On the other hand, our study demonstrated that the creative drama method increased the self-esteem of students, and this result is likely to affect the conflict resolution style positively in the long run. Our study findings suggest that in order to evaluate the effect of creative drama method on the conflict resolution styles utilized by midwifery undergraduates, it is necessary to implement longer education programs and to evaluate the post-education evaluation with long-term prospective studies.

Using creative drama in the education process of midwifery undergraduates who will be at the core of maternal and child healthcare services could affect positively the quality of midwifery education through the introduction of theoretical knowledge into practice, the in-depth exploring of knowledge, offering personal development in addition to vocational education, providing learning based on experience, and acquiring communication processes through lifelike interaction^35-37^. Findings obtained from our study support this view. Besides, creative drama could be an effective learning/experience method for midwifery and nursing students to deal with common communication difficulties encountered while putting theoretical knowledge into practice^30^. Furthermore, considering that health science educators have difficulties in balancing the process of providing students with practical skills^27,30,38^, the creative drama method could raise the quality of education.

## CONCLUSIONS

The creative drama method is effective in developing the communication skills and self-esteem of midwifery undergraduates. Applying creative drama in midwifery departments as a training method could be a useful tool for developing cognitive skills that are very difficult to acquire, and could facilitate the implementation of theoretical knowledge acquired within vocational courses. It is recommended that further studies are conducted to analyze the effectiveness of creative drama on different cognitive skills that midwives should have and to identify the effects of using the creative drama technique in vocational courses.

## Data Availability

The data supporting this research are available from the authors on reasonable request.
